# A novel alcohol dehydrogenase in the hyperthermophilic crenarchaeon *Hyperthermus butylicus*

**DOI:** 10.1002/mlf2.12126

**Published:** 2024-06-28

**Authors:** Ching Tse, Kesen Ma

**Affiliations:** ^1^ Department of Biology University of Waterloo Waterloo Ontario Canada

**Keywords:** butanol, hyperthermophile, *Hyperthermus butylicus*, novel alcohol dehydrogenase, thermostability

## Abstract

*Hyperthermus butylicus* is a hyperthermophilic crenarchaeon that produces 1‐butanol as an end product. A thermostable alcohol dehydrogenase (ADH) must be present in *H. butylicus* to act as the key enzyme responsible for this production; however, the gene that encodes the ADH has not yet been identified. A novel ADH, HbADH2, was purified from a cell‐free extract of *H. butylicus*, and its characteristics were determined. The gene that encodes HbADH2 was demonstrated to be *HBUT_RS04850* and annotated as a hypothetical protein in *H. butylicus*. HbADH2 was found to be a primary–secondary ADH capable of using a wide range of substrates, including butyraldehyde and butanol. Butyraldehyde had the highest specificity constant, calculated as *k*_c__at_/*K*_m_, with *k*_cat_ and apparent *K*_m_ values of 8.00 ± 0.22 s^−1^ and 0.59 ± 0.07 mM, respectively. The apparent *K*_m_ values for other substrates, including ethanol, 1‐propanol, 2‐propanol, butanol, acetaldehyde, propanal, and acetone, were 4.36 ± 0.42, 4.69 ± 0.41, 3.74 ± 0.46, 2.44 ± 0.30, 1.27 ± 0.18, 1.55 ± 0.20, and 0.68 ± 0.04 mM, respectively. The optimal pH values for catalyzing aldehyde reduction and alcohol oxidation were 6.0 and 9.0, respectively, while the optimal temperature was higher than 90°C due to the increase in enzymatic activity from 60°C to 90°C. Based on its substrate specificity, enzyme kinetics, and thermostability, HbADH2 may be the ADH that catalyzes the production of 1‐butanol in *H. butylicus*. The putative conserved motif sites for NAD(P)^+^ and iron binding were identified by aligning HbADH2 with previously characterized Fe‐containing ADHs.

## Impact statement

*Hyperthermus butylicus* is a hyperthermophile reported to produce a significant amount of 1‐butanol. A novel thermostable alcohol dehydrogenase (ADH) from *H. butylicus* has been characterized to be a new type of ADH. Through the study of the essential enzyme, HbADH2, in *H. butylicus*, we have gained further insights into the butanol production pathway in *Crenarchaeota*. The discovery of this novel thermostable ADH provides the possibility of using it as a biocatalyst for biobutanol production, a process that has regained interest in the wake of increasing renewable energy demands worldwide. Due to its similar thermodynamic and physical properties to gasoline, biobutanol is a promising fuel alternative that can be used in traditional vehicles.

## INTRODUCTION

*Hyperthermus butylicus* is a heterotrophic sulfur‐reducing archaeon belonging to *Crenarchaeota* ^1^. *H. butylicus*, first isolated from the sea floor on the coast of Sao Miguel, Azores, is a neutrophile with a sharp optimal pH of 7.0 and grows optimally at a salt concentration of 17 g/l NaCl^1^. It has a broad temperature range for growth—between 80°C and 108°C—with optimal growth temperatures between 95°C and 106°C^1^. *H. butylicus* can utilize peptide mixtures as carbon and energy sources, and all genes encoding the enzymes of the gluconeogenesis pathway have been found in its genome^1^, ^2^. *H. butylicus* metabolizes energy primarily through fermentation while using elemental sulfur (S°) and H_2_ as additional energy sources to support its growth^1^.

Gas chromatography–mass spectroscopy and nuclear magnetic resonance spectroscopy have been used to determine the major metabolic end products of the fermentation of *H. butylicus* ^1^. The end products detected include 0.67 mM of 1‐butanol; a similar amount of acetic, propionic, and phenylacetic acid; a small amount of hydroxyphenylacetic acid; and trace amounts of propylbenzene, acetophenone, and hydroxyacetophenone^1^. The production of butanol was identified through distillation and the determination of the boiling point of 1‐butanol^1^. The nature of the end products suggests that they arose from the fermentation of peptides^3^, ^4^. However, the metabolic pathway of 1‐butanol has not yet been identified in *H. butylicus*. The genome of *H. butylicus* lacks a homologue of the bacterial acetoacetate‐butyrate/acetate‐CoA transferase, which is essential for 1‐butanol production in most *Clostridium* species^5^. Although few studies have investigated the metabolic pathway of alcohol production in hyperthermophiles, it is unquestionable that alcohol dehydrogenase (ADH)—specifically, butanol dehydrogenase for use in butanol production—is involved as an essential enzyme.

The identification of the ADH responsible for 1‐butanol production in *H. butylicus* represents an initial step toward understanding the physiological importance of this pathway. One gene in the genome of *H. butylicus*, *HBUT_RS02160* (previous locus tag: Hbut_0414), was found to encode an amino acid sequence with a conserved domain corresponding to that of the zinc‐dependent ADH‐like family. The gene was cloned and overexpressed in *Escherichia coli* ^6^. However, the biophysical and biochemical characteristics of the recombinant enzyme (HbADH1) encoded by *HBUT_RS02160* suggest that *HBUT_RS02160* is unlikely to encode the ADH responsible for catalyzing the production of 1‐butanol in *H. butylicus* ^6^. Many microorganisms, including hyperthermophiles, contain multiple ADHs^7^. Two well‐characterized examples are various putative ADHs from *Sulfolobus solfataricus* and two different types of ADHs from *Pyrococcus furiosus* ^8^, ^9^, ^10^, ^11^, ^12^. It is notable that the properties of thermophilic ADHs might be related to their phenotypic origins^7^, ^13^. While most marine hyperthermophiles have at least one iron‐containing ADH, most terrestrial ones have zinc‐containing ADHs as well as other types^7^, ^13^. Given that *H. butylicus* was isolated from a marine biotope^1^, it is highly probable that it harbors iron‐containing ADH. However, no gene annotations have indicated that there is an iron‐containing ADH in the genome sequence of *H. butylicus*. Our present study was to first detect ADH activity in a cell‐free extract (CFE) of *H. butylicus*, purify this novel ADH, and then identify the gene that encodes it. The biophysical and biochemical characteristics of this novel ADH (HbADH2) were studied to predict its physiological function in *H. butylicus*.

## RESULTS

### Purification of novel HbADH2 from *H. butylicus*

**Figure 1:**
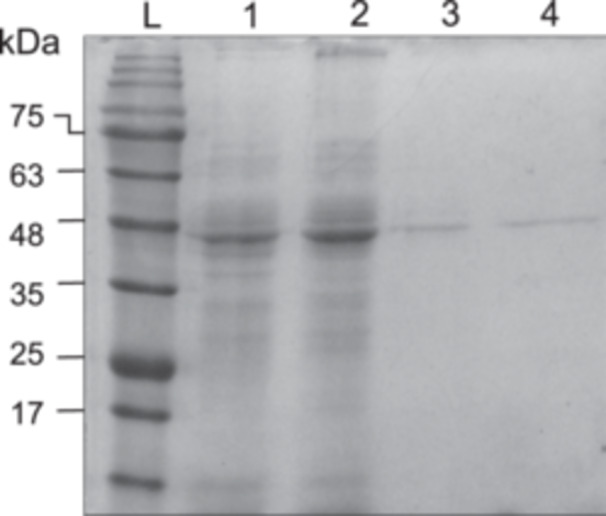
Purified HbADH2 from *Hyperthermus* *butylicus* on sodium dodecyl sulfate–polyacrylamide gel electrophoresis (12.5%). L, a low‐molecular‐weight protein marker; lane 1, *H. butylicus* cell‐free extract (15.7 µg total protein loaded); lane 2, combined fractions with HbADH2 activity eluted from the DEAE‐Sepharose column (20.8 µg total protein loaded); lane 3, combined fractions with HbADH2 activity eluted from the HAP column (2.63 µg total protein loaded); lane 4, purified HbADH2 from phenyl‐Sepharose (1.67 µg total protein loaded).

ADH activity in a CFE of *H. butylicus* was initially confirmed using 1‐butanol and butyraldehyde as substrates. The ADH(s) from the CFE demonstrated activity toward both NADP(H) and NAD(H), whereas the ADH encoded by *HBUT_RS02160* is an NAD(H)‐dependent enzyme^6^. This observation suggests the presence of a novel ADH in *H. butylicus* that could have the potential to catalyze the production of 1‐butanol in this organism, and yet, it has not been annotated or characterized thus far. The novel ADH discovered in this study, which we call HbADH2, was purified through a series of chromatography columns (Table S1). A CFE containing 41 U of HbADH2 activity was first loaded onto a diethylaminoethyl (DEAE)‐Sepharose column. However, most of the other *H. butylicus* proteins bound to the DEAE‐Sepharose column along with the HbADH2, resulting in only 1.1‐fold purification. Subsequently, HbADH2 was found to strongly bind to a hydroxyapatite (HAP) column and eluted after most of the other *H. butylicus* proteins. This resulted in a 4.3‐fold increase in purity. The partially purified HbADH2 was further purified using a phenyl‐Sepharose column. Since HbADH2 was weakly bound to the column, it was eluted before the other *H. butylicus* proteins and ended up homogeneous as a result. The purity of HbADH2 was then verified using sodium dodecyl sulfate‐polyacrylamide gel electrophoresis (SDS‐PAGE) (Figure 1). The yield of the purification was about 52%. The purified HbADH2 was stored under anaerobic conditions at −20°C with 5 psi of nitrogen gas for further characterization experiments.

### Protein concentration‐dependent HbADH2

To determine the appropriate protein concentration for characterizing the purified HbADH2, different concentrations were tested by initiating enzyme activity within them. Protein amounts ranging from 6 to 30 μg were examined for their respective activity levels. The activity of HbADH2 increased proportionally as more of it was added to the assay system (Figure S1). However, beyond 20 µg, the proportional increase in activity ceased. Hence, the specific activity of HbADH2 was recorded as 8.2 ± 0.72 U/mg at a quantity of ≤20 µg and decreased beyond 20 µg, which may be due to the saturation of the enzyme at higher concentrations (>20 µg). Thus, 15 μg of HbADH2 was selected as the appropriate amount for subsequent enzyme assays, as it was within the range required to achieve the highest determinable specific activity.

### Temperature‐dependent activity and thermostability of HbADH2

**Figure 2:**
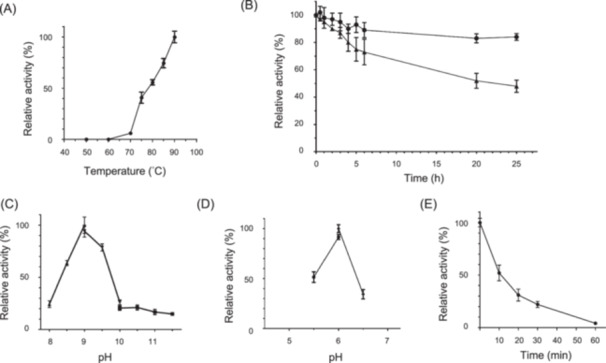
Enzymatic properties of HbADH2 from *Hyperthermus* *butylicus*. (A) Temperature dependency of HbADH2 activity. Activity was measured under standard assay conditions, with temperatures varying from 50°C to 90°C. A relative activity of 100% was defined as the activity measured at 90°C, which was 13.6 U/mg, with 60 mM of 1‐butanol as the substrate. (B) Thermostability of HbADH2 with incubation of HbADH2 at 85°C (filled circles) and 95°C (filled triangles). The relative activity of 100% refers to the initial HbADH2 activity without heat treatment (8.2 U/mg). Enzyme assays were performed using 60 mM of 1‐butanol as the substrate at 80°C. (C) pH dependency of HbADH2 activity in alcohol oxidation. Activity was measured with 60 mM of 1‐butanol and in 100 mM of 4‐(2‐Hydroxyethyl)‐1‐piperazinepropanesulfonic acid (filled triangles), glycine (filled inverted triangles), and 3‐(Cyclohexylamino)‐1‐propanesulfonic acid (filled squares). A relative activity of 100% equals 8.2 U/mg of alcohol oxidation activity. The pH values of the buffers were measured at room temperature. (D) pH dependency of HbADH2 activity in aldehyde/ketone reduction. Activity was measured with 60 mM of butyraldehyde and buffers (100 mM) of citrate (filled circles) and piperazine‐N,N′‐bis(2‐ethanesulfonic acid) (filled diamonds). A relative activity of 100% equals 11.7 U/mg of aldehyde reduction activity. The pH values of the buffers were measured at room temperature. (E) Oxygen sensitivity of HbADH2. The activity was measured in the presence of 2 mM of dithiothreitol and 2 mM of sodium dithionite (SDT). A relative activity of 100% equals the activity of HbADH2 before exposure to oxygen, with 60 mM of 1‐butanol as the substrate (8.2 U/mg). All data are presented in the form of mean ± standard derivation (*n* = 2).

The optimal temperature for promoting enzymatic activity in HbADH2 was determined by assessing its performance across a temperature range of 50°C to 90°C, with 1‐butanol serving as the substrate. The results demonstrated that the enzyme activity increased steadily from 60°C to 90°C, with no activity observed below 60°C (Figure 2A). The optimal temperature is likely to be greater than 90°C because the optimal growth temperature of *H. butylicus* is 95°C^1^, and enzyme activity could not be measured at temperatures above 90°C because the instruments were unable to increase the temperature to these levels. The relative enzymatic activity of HbADH2 was approximately 50% and 75% at 75°C and 85°C, respectively, compared to the enzymatic activity at 90°C (Figure 2A).

The thermostability of HbADH2 was evaluated at its optimal growth temperature, 95°C, and at 85°C. HbADH2 demonstrated exceptional thermostability at 85°C, with its relative activity decreasing to only 90% after 25 h of incubation (Figure 2B). Moreover, when incubated at 95°C, the enzyme displayed a half‐life (*t*_1/2_) of approximately 25 h, with a more rapid decrease in activity observed during the first 5 h of incubation. Specifically, the residual activity declined to 75% of the initial level after 5 h of incubation at 95°C (Figure 2B). A semi‐log plot of residual activity versus heating time showed a linear trend at both 85°C and 95°C (Figure S2). The slopes of these lines indicated that the inactivation rate constant at 95°C was about four times higher than that at 85°C.

### pH‐dependent activity of HbADH2

The optimal pH levels for reduction and oxidation reactions with HbADH2 were determined using a range of 100‐mM buffers with pH values spanning from 5.5 to 6.5 and 8.0 to 11.5, respectively. The resulting data indicate that the optimal pH for the oxidation of 1‐butanol is 9.0 (Figure 2C), while the optimal pH for the reduction of butyraldehyde is 6.0 (Figure 2D). Notably, the reduction activity showed a pronounced optimum pH at 6.0, whereas the oxidation activity displayed a sharp decline when the pH exceeded 9.5.

### Oxygen sensitivity of HbADH2

The oxygen sensitivity of HbADH2 was evaluated by exposing the enzyme to an aerobic environment for 1 h, with residual activity measured at various intervals. The results revealed that HbADH2 was highly sensitive to oxygen exposure, with residual activity decreasing to 50% after a 10‐min exposure to oxygen in the presence of 2 mM of dithiothreitol (DTT) and 2 mM of sodium dithionite (SDT) (Figure 2E). After 30 min of exposure to oxygen, the residual activity was less than 25%. A semi‐log plot of residual activity versus duration of exposure to oxygen demonstrated a linear relationship, with an inactivation constant of 0.0524 min^−1^ (Figure S3).

### Substrate specificity and enzyme kinetics

The substrate specificity of HbADH2 was assessed using a range of alcohols, aldehydes, and ketones (Table 1). The highest oxidation activity was observed with 1‐butanol, while the highest reduction activity was demonstrated with butyraldehyde, the corresponding aldehyde. In the oxidation reaction, HbADH2 was capable of oxidizing primary and secondary alcohols, except methanol, indicating that it is a primary–secondary ADH (Table 1).

**Table 1:** Substrate specificity in oxidation and reduction reactions of HbADH2 from *Hyperthermus* *butylicus*.

Substrate alcohol (oxidation)^a^	Relative activity (%)
Methanol	0
Glycerol	0
Ethanol	53
1‐propanol	64
1‐butanol	100^b^
1‐pentanol	57
1‐hexanol	20
1‐heptanol	6
1‐octanol	2
2‐propanol	48
2‐butanol	75
2‐phenylethanol	44
1,3‐propanediol	8
1,2‐butanediol	10
1,3‐butanediol	9
2,3‐butanediol	3
1,4‐butanediol	14
1,5‐pentanediol	7
2,4‐pentandiol	2

Aldehydes/ketones (reduction)^c^	Relative activity (%)
Benzaldehyde	49
Acetaldehyde	78
Propanal	20
Butyraldehyde	100^d^
Acetone	45
Butanone	3.9

^a^ Enzyme assays were performed under standard conditions with a pH of 9.0°C and at 80°C with various substrates. ADH activity was initiated through the addition of purified HbADH2. Oxidation activity was measured with 0.4 mM of NADP^+^.

^b^ A relative activity of 100% in alcohol oxidation was equated with a value of 8.4 ± 0.2 U/mg.

^c^ Enzyme assays were performed under standard conditions with a pH of 6.0°C and at 80°C with various substrates. ADH activity was initiated through the addition of purified HbADH2. Reduction activity was measured with 0.2 mM of NADPH.

^d^ A relative activity of 100% in aldehyde/ketone reduction was equated with a value of 11.7 ± 0.4 U/mg.

The activity toward primary alcohols was greater than that toward secondary alcohols. The relative activity for diols was considerably lower than that for alcohols, with slightly higher activity observed for primary diols than for secondary diols. HbADH2 showed no activity toward the polyol‐glycerol that we examined. In the reduction reactions, HbADH2 displayed the ability to reduce a range of aldehydes and ketones (Table 1), with higher relative activity observed for acetone than for propanal and for butyraldehyde than for butanone. Generally, there was more activity with aldehydes than with ketones.

The apparent *K*_m_ values for the substrates in the reduction reaction were approximately four times lower than those for the corresponding alcohols in the oxidation reaction (Table 2). Specifically, the *K*_m_ value for butyraldehyde was about four times lower than that for 1‐butanol, and the *K*_m_ value for acetone was 5.5 times lower than that for 2‐propanol. Among the examined substrates, the lowest *K*_m_ value was found for butyraldehyde. The specificity constant (*k*_cat_/*K*_m_) for butyraldehyde was the highest (13,560 ± 3140 s^−1 ^M^−1^), at approximately 2.5 times higher than that for acetaldehyde, and 4.8 times higher than that for 1‐butanol. The specificity constant for acetone was 8.7 times higher than that for 2‐propanol. Notably, the specificity constants were higher for aldehyde and ketone reduction than for alcohol oxidation (Table 2). These catalytic properties suggest that HbADH2 could play a crucial role in the reduction of butyraldehyde in vivo.

**Table 2:** Kinetic parameters of HbADH2 from *Hyperthermus* *butylicus*.

Substrate (mM)^a^	Cofactor (mM)	Apparent *K*_m_ (mM)	*k*_cat_ (s^−1^)	*k*_cat_ */K*_m_ (s^−1^M^−1^)
Ethanol (0.36–48.58)	NADP (0.4)	4.36 ± 0.42	4.00 ± 0.13	915.4 ± 308
1‐Propanol (0.36–48.58)	NADP (0.4)	4.69 ± 0.41	4.40 ± 0.12	938.4 ± 293
2‐Propanol (0.36–48.58)	NADP (0.4)	3.74 ± 0.46	3.47 ± 0.13	927.8 ± 413
Butanol (0.36–48.58)	NADP (0.4)	2.44 ± 0.30	6.84 ± 0.24	2,803 ± 814
Acetaldehyde (0.36–13.82)	NADPH (0.2)	1.27 ± 0.18	7.00 ± 0.31	5,512 ± 1720
Propanal (0.36–26.43)	NADPH (0.2)	1.55 ± 0.20	1.79 ± 0.07	1,155 ± 338
Acetone (0.36–26.43)	NADPH (0.2)	0.68 ± 0.04	3.84 ± 0.06	8,074 ± 1520
Butyraldehyde (0.36–13.82)	NADPH (0.2)	0.59 ± 0.07	8.00 ± 0.22	13,560 ± 3140

^a^ Different concentrations of alcohol (0.36, 0.72, 1.08, 1.44, 2.16, 2.87, 3.58, 7.08, 13.82, 26.43, and 48.58 mM) were used to measure the parameters of alcohol oxidation kinetics, and different concentrations of aldehydes/ketones (0.36, 0.72, 1.08, 1.44, 2.15, 2.87, 3.58, 7.08, 13.82, and 26.43 mM) were used to measure the parameters of aldehyde/ketone reduction kinetics. Data are presented in the form of mean ± standard deviation.

### Identification of the gene encoding HbADH2 and sequence analysis

The band corresponding to the novel HbADH2 protein on SDS‐PAGE (Figure 1) was cut out and sent to the University of Alberta for peptide identification via mass spectrometry. The mass spectrometer identified five proteins in the band (Table S2). In their denatured form, proteins of similar sizes cannot be separated out using SDS‐PAGE. Instead, we recommend a size‐exclusion column, which can effectively isolate proteins based on their molecular weight in the native form. However, only about 2.6 mg of protein was eluted from HbADH2 in the phenyl‐Sepharose column. Further purification of HbADH2 using the size‐exclusion column was not carried out to prevent further dilution of the HbADH2 and ensure that enough proteins were included when running SDS‐PAGE to produce a protein band that would allow the peptides to be identified. Four proteins have been identified using a mass spectrometer and annotated as gene encoding enzymes in the *H. butylicus* genome (Table S2). *HBUT_RS04595* (previous locus tag: Hbut_0873) was annotated as a 4‐aminobutyrate aminotransferase with an e‐value of 2.49 e^−113^, *HBUT_RS08315* (previous locus tag: Hbut_1589) as an alanine‐glyoxylate aminotransferase family protein with an e‐value of 1.69 e^−157^, *HBUT_RS02620* (previous locus tag: Hbut_0503) as a type‐III ribulose bisphosphate carboxylase with an e‐value of 0, and *HBUT_RS02345* (previous locus tag: Hbut_0451) as an Xaa‐Pro peptidase family protein with an e‐value of 1.53. The low e‐values strongly indicate the presence of these proteins in our sample, but surely, none of them could be an ADH because they are not oxidoreductases. The protein encoded by *HBUT_RS04850* (previous locus tag: Hbut_0924) has the highest identification score according to the mass spectrometer data (Table S2) and was annotated as a hypothetical protein. The high probability that our novel ADH, HbADH2, is encoded by gene *HBUT_RS04850* is reinforced by the protein identification scores and number of unique peptides identified, which suggests an approximate purity of 80% (Table S2). The nucleotide sequence and its corresponding encoded amino acid sequence were further analyzed and compared with other annotated ADHs from genomes and proteomes of hyperthermophiles available from NCBI^14^ (Table S3, Figure S4).

## DISCUSSION

*H. butylicus* is a hyperthermophile that produces a considerable amount of 1‐butanol as an end product along with acetic, propionic, and phenylacetic acid^1^. The identification of a butanol production pathway in *H. butylicus* is of great scientific interest. However, an analysis of the genome of *H. butylicus* did not reveal any homologues of key enzymes in the butanol production pathway of *Clostridium*, including butyryl‐CoA dehydrogenase, β‐hydroxybutyryl‐CoA dehydrogenase, acetoacetyl‐CoA:acetate:CoA‐transferase, acetoacetyl‐CoA:butyrate:CoA transferase, and butyraldehyde dehydrogenase^6^. *H. butylicus* exclusively uses peptides as growth substrates, which suggests that it may use the butanol production pathway in *Saccharomyces cerevisiae* ^1^. However, homologues of most corresponding enzymes in the pathway were not found in the genome of *H. butylicus*, including malate synthase, α/β‐isopropylmalate dehydrogenase, α‐isopropylmalate synthase, α‐isopropylmalate isomerase, pyruvate decarboxylase, and 2‐ketoacid decarboxylase. Although homologues of two enzymes, glycine oxidase and threonine dehydratase, were found in the genome, they are also used for amino acid metabolism, and their roles in butanol production remain unclear^15^, ^16^. The pathway for butanol production in *H. butylicus* is unknown, but it is evident that ADH plays a critical role in the reduction of aldehyde to alcohol in vivo, and its presence in *H. butylicus* is essential for 1‐butanol production.

The approach used in this study to predict the ADH responsible for 1‐butanol production in *H. butylicus* was to purify a novel ADH from a CFE. Gene *HBUT_RS04850* was identified as the one that encodes this novel purified ADH, which we call HbADH2 (Table S2)*.* This gene comprises 1104 bp with 367 deduced amino acid residues, and the protein has a calculated molecular weight of 40,694 Da. It has been annotated as a hypothetical protein, but no conserved domain has been identified. To further investigate the potential function of *HBUT_RS04850*, nucleotide and protein blast searches were conducted. While the nucleotide searches (MegaBLAST and BLASTN) against all genomes returned no significantly similar matches, the protein blast search (Blastp, Protein–Protein BLAST) for similar amino acid sequences revealed significant parallels with four hypothetical proteins from hyperthermophiles (Table S3). However, no conserved domains could be identified in these proteins, even though they are over 30% identical to the amino acid sequence encoded by *HBUT_RS04850*.

Since nontarget searches were not able to identify a conserved domains corresponding to that of ADH, the gene *HBUT_RS04850* was searched against the genomes of related hyperthermophiles. A BLASTN search against the genome of *Desulfurococcus mucosus* DSM 2162 (taxid: 765177), a species from the phylum *Crenarchaeota*, returned a significant result. It showed that *HBUT_RS04850* had the lowest e‐value, 7 e^−4^, with an iron‐containing ADH in the genome of *D. mucosus*. Conversely, a BLASTN search using the gene (*Desmu_0302*) that encodes this iron‐containing ADH from *D. mucosus* against the genome of *H. butylicus* DSM 5456 (taxid: 415426) returned a significant similarity to *HBUT_RS04850* with the lowest e‐value, 9 e^−4^.

**Figure 3:**
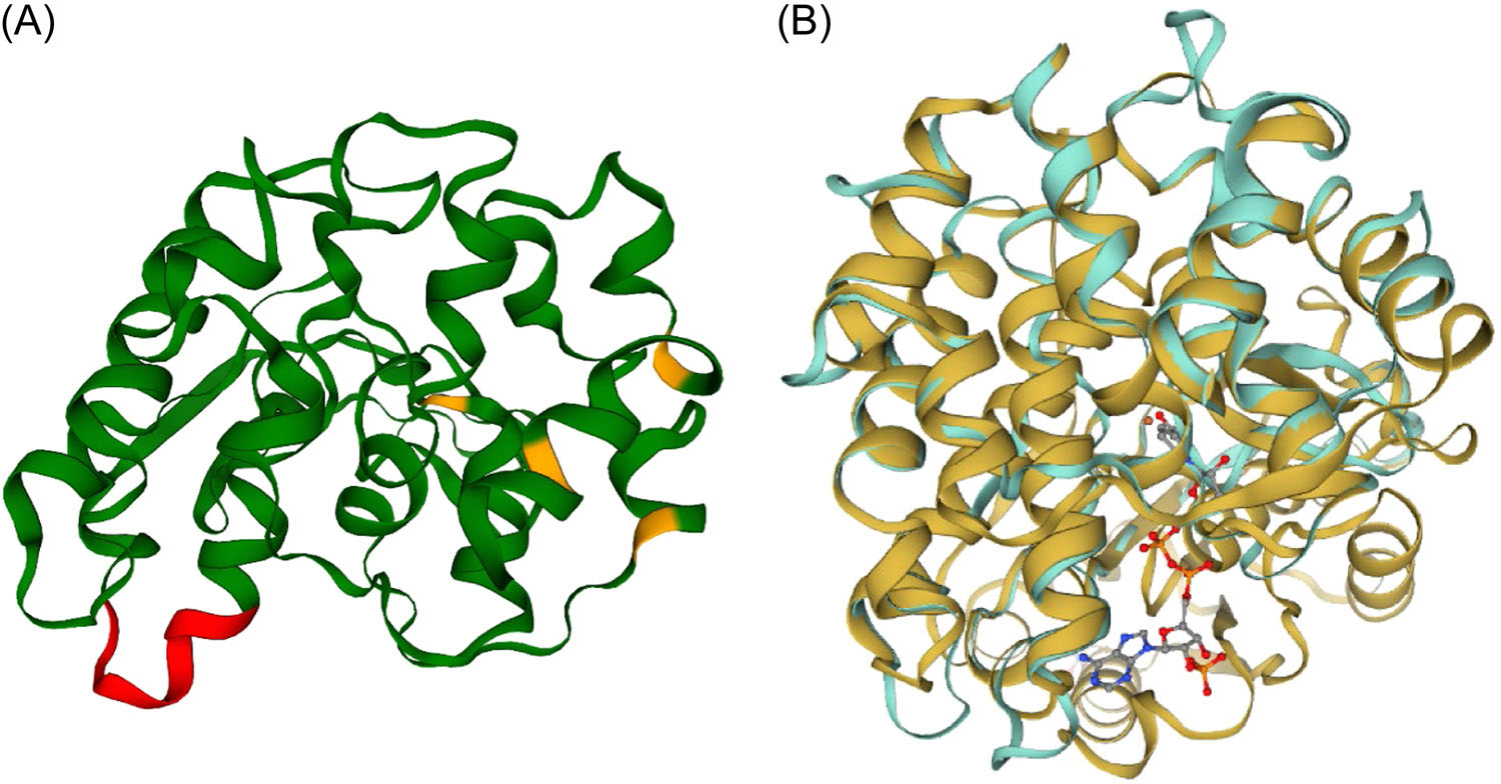
Predicted three‐dimensional (3D) tertiary structure of the HbADH2 monomer. (A) 3D structural modeling was run on the Swiss Model server using an iron‐containing ADH from *Thermotoga maritima* (TM0920; Protein Data Bank [PDB] number: 1O2D) as the template. Residues in red indicate putative NADP^+^ binding sites; residues in yellow indicate putative iron binding sites. (B) Superimposition of the predicted 3D tertiary structure of the HbADH2 monomer (in turquoise) onto the 3D tertiary structure of iron‐containing ADH from *T. maritima* (TM0920; PDB number: 1O2D) (in gold) with amino acids for the NAD(P)^+^ binding sites.

To elucidate the potential function of HbADH2 by examining its amino acid sequence, the amino acid sequence encoded by *HBUT_RS04850* was aligned with the sequences of the other characterized iron‐containing ADHs. These ADHs belong to the same protein family, which has conserved domains for NAD(P)^+^‐ and iron metal‐binding motifs. Through the comparison, a putative cofactor NAD(P)^+^‐binding motif (G_64_G_65_G_66_XXI_69_) was discovered in the amino acid sequence encoded by *HBUT_RS04850* in *H. butylicus* (Figure S4). Additionally, the putative iron‐binding motif in its amino acid sequence is only slightly different (Figure S4). Specifically, the three histidine residues (H_130_H_223_H_231_) are conserved, with only the aspartic acid being replaced, with glutamic acid (E_195_), in *H. butylicus*. This indicates that the two amino acids have highly similar properties. Moreover, the amino acid sequence from *H. butylicus* is about 20% identical to those of the ADHs. The greatest similarity was found between ADH from *Thermococcus litoralis* (EHR78112.2) and ADH from *Thermococcus paralvinellae* ES‐1 (ACK56133.1), with an identity of around 21%. The diversity of amino acid sequences in iron‐containing ADHs and the low rate of sequence similarity may explain why *HBUT_RS04850* has not yet been annotated as an ADH in the genome. The three‐dimensional structure of HbADH2 was predicted by using the crystal structure of an iron‐containing ADH from *Thermotoga maritima* (TM0920) as a model (Figure 3A). The family of iron‐containing ADHs typically consists of two characteristic domains: an α/β‐dinucleotide‐binding N‐terminal domain and an all‐α‐helix C‐terminal domain, with a deep cleft separating them^17^. The predicted structure of HbADH2 shows characteristics similar to those of these domains, as demonstrated by the superimposition on the crystal structure of *T. maritima* ADH (Figure 3B). However, further research is necessary to validate the accuracy of the structural modeling prediction.

The enzyme encoded by *HBUT_RS04850*, named HbADH2, was found to be a primary–secondary ADH that shows higher relative activity toward primary alcohols than toward secondary alcohols. The determined apparent *K*_m_ values for aldehydes were lower than those for alcohols, indicating a preference for catalyzing alcohol production. HbADH2 demonstrated the highest specificity constant (*k*_cat_/*K*_m_) with butyraldehyde, which is converted into 1‐butanol by ADH. HbADH2 is highly oxygen‐sensitive, probably because it is an iron‐containing ADH. HbADH2 is remarkably thermostable, and its activity continues to increase up to 90°C. Given that the optimal growth temperature for *H. butylicus* is between 95°C and 106°C^1^, the thermostability of HbADH2 suggests that it is functionally expressed in *H. butylicus*. HbADH1 and the other ADHs characterized in *H. butylicus* shared a preference for primary alcohols and lower *K*_m_ values for aldehydes than for alcohols^6^. However, HbADH1 is a zinc‐containing, NAD^+^‐dependent, and oxygen‐insensitive ADH, and the optimal pH values for reduction and oxidation reactions are 5 and 8.5, respectively. HbADH1 demonstrated the highest specificity constant with propanal^6^, but its specificity constant with butyraldehyde (1120 s^−1 ^M^−1^)^6^ is more than 10 times lower than that of HbADH2 (13,560 s^−1 ^M^−1^). In addition, HbADH1 has an optimal temperature of 60°C and a half‐life (*t*_1/2_) of about 3 h at 60°C^6^. Their differences in catalytic properties, oxygen sensitivity, thermostability, and optimum pH strongly suggest that HbADH2, rather than HbADH1, functions as the ADH involved in butanol production in *H. butylicus*. Future research on the expression levels of HbADH1 and HbADH2 would provide further insights into their physiological functions.

In conclusion, the novel ADH purified in this study, HbADH2, is encoded by *HBUT_RS04850*. Putative cofactor NAD(P)^+^‐ and iron‐binding motif sites were identified, which suggests that HbADH2 is an NAD(P)H‐dependent iron‐containing ADH. The sequence similarity between HBUT_RS04850 and other ADHs was quite low, revealing the former to be a novel type of iron‐containing ADH in hyperthermophiles. Based on the biochemical and biophysical characteristics of HbADH2, we propose that HbADH2 may be the ADH responsible for the production of 1‐butanol in *H. butylicus*. Further investigations involving gene knockout and gene complementation experiments with *HBUT_RS04850* are necessary to confirm whether HbADH2 is indeed the ADH responsible for butanol production in *H. butylicus*. Moreover, studying the regulation of HbADH2 expression and its activity in *H. butylicus* would provide further insights into the metabolic pathway that leads to 1‐butanol production in *Crenarchaeota*.

## MATERIALS AND METHODS

### Microorganisms and chemicals

*H. butylicus* (DSM 5456) from the Deutsche Sammlung von Mikroorganismen and Zellkulturen, was used for this study. All chemicals were commercially available.

### Growth of *H. butylicus*

*H. butylicus* was grown in the medium as described by Zillig et al.^1^ The trace element solution was prepared according to the DSMZ formula, with modifications from Balch^18^.

### Preparation of CFE

All steps for the preparation of CFE were performed anaerobically. Frozen cells of *H. butylicus* were transferred into a serum bottle filled with N_2_ gas and the bottle was immediately sealed using a gray butyl stopper, and capped with an aluminum seal. The bottle was then immediately degassed for 30 min and pressurized with 3 psi of N_2_ gas. The lysis buffer contained 50 mM Tris‐HCl at pH 7.5, 2 mM SDT, 2 mM DTT, 0.1 mg/ml lysozyme, and 0.01 mg/ml DNase I (Sigma), which was also made anaerobically. The ratio of the anaerobic lysis buffer used to the weight of the cells was 5:1. The lysis buffer needed was then anaerobically transferred to the serum bottle containing the cells using a syringe, and the cell suspension obtained was further stirred using a magnetic stirrer for 2 h at 37°C. The cell suspension was then treated using a French Press cell (Thermo Fisher Scientific) and the *H. butylicus* cells were lysed at 25,000 psi. The lysis mixture was the crude cell extract, which was then centrifuged at 8000 rpm for 15 min at 4°C to remove cell debris. The supernatant was the CFE that was then stored anaerobically in a serum bottle for further use.

### Assay of alcohol dehydrogenase

The catalytic activity of *H. butylicus* novel ADH (HbADH2) was determined at 80°C by monitoring the selected substrate‐dependent absorbance change of NADP(H) at 340 nm (*ε*_340_ = 6.3 mM^−1 ^cm^−1^)^19^. If not specified, all enzyme assays were carried out in duplicate. For measuring the alcohol oxidation, the assay mixture (2 ml) contained 60 mM 1‐butanol and 0.4 mM NADP^+^ in the buffer of 100 mM EPPS [4‐(2‐hydroxyethyl)‐1‐piperazinepropanesulfonic acid, pH 9.0]. For the reduction of ketone/aldehyde, the assay mixture (2 ml) contained 30 mM butyraldehyde and 0.4 mM NADP(H) in 100 mM PIPES [piperazine‐N,N′‐bis(2‐ethanesulfonic acid), pH 6.0]. The addition of the appropriate amount of purified enzyme (15 μg) initiated the enzyme assay. One unit of activity is defined as 1 μmol of NADPH formation or oxidation per min.

### Purification of the novel HbADH2

AKTA^TM^ Fast Performance Liquid Chromatography (FPLC), a liquid chromatography system with a P‐920 pump (Amersham Pharmacia Biotech), was used for the purification of the enzyme under anaerobic conditions at room temperature. Buffer A contained 50 mM Tris‐Base, 5% (v/v) glycerol, 2 mM SDT, and 2 mM DTT. The buffer was filtered to remove any impurity using a vacuum filtration device and made anaerobically. Three columns were used in series for completing the purification of the HbADH2.

The CFE was loaded onto a DEAE‐Sepharose column (3.5 cm × 5 cm, column volume [CV] 35 ml) that was equilibrated with 2 CV of buffer A at a flow rate of 2 ml min^−1^. Buffer B was made of buffer A plus 2 M NaCl. The proteins bound to the column were eluted using a gradient of 3 CV buffer B from 5% to 50% and the novel HbADH2 was eluted when 0.55 M of NaCl in buffer B was applied to the column. Fractions containing the HbADH2 activity were combined and loaded onto a hydroxyapatite column (HAP, 2.6 cm × 12 cm, CV 50 ml) at a flow rate of 1.0 ml min^−1^. The column was applied with a gradient of buffer C (0.32 M potassium phosphate dibasic and 0.18 M potassium monobasic in buffer A) and HbADH2 started to elute from the column when 0.5 M potassium phosphate buffer was applied to the column. Fractions containing the HbADH2 activity were then pooled and applied to a phenyl‐Sepharose column (5 cm × 10 cm) that was equilibrated with 2.0 M (NH_4_)_2_SO_4_ in buffer A overnight at a flow rate of 0.5 ml min^−1^. HbADH2 started to elute when 0.8 M of (NH_4_)_2_SO_4_ was applied. Fractions with HbADH2 activity were desalted and concentrated by ultrafiltration using an Ultracel PL 10 membrane (EMD Millipore Ultracel) and collected in 6 ml serum bottles. The purity of the fractions containing HbADH2 activity was verified using SDS‐PAGE based on the methods described by Laemmli^20^. The molecular mass of the enzyme subunit was estimated by using SDS‐PAGE and the protein marker Extended PS 13 (5–245 kDa; GeneON).

### Determination of catalytic properties of the HbADH2

The optimal pH values for alcohol oxidation and aldehyde/ketone reduction of the purified HbADH2 were measured by enzyme assay of 1‐butanol oxidation and butyraldehyde reduction over a range of 5.5–11.5. The standard enzyme assay was performed with 100 mM buffer at different pH values at 80°C. The buffers (100 mM) used were citrate (pH 5.5–6.0), PIPES (pH 6.0–6.5), EPPS (8.0–9.0), glycine (9.0–10.0), and CAPS (10.0–11.5).

The temperature‐dependent HbADH2 activity was measured at temperatures from 50°C to 90°C using the standard enzyme assay described above. Enzyme thermostability was determined by incubating the enzyme in sealed serum bottles at 85°C and 95°C. Residual activities were measured after each specified time interval of the incubation, and then they were compared to the initial activity without incubation at high temperatures.

The effect of oxygen on enzyme activity was investigated by determining the residual activity after exposure of the purified HbADH2 in the presence of 2 mM DTT and 2 mM SDT to air at room temperature under stirring. The residual activities of each sample at each specified time interval were determined using the standard assay as described above.

Substrate specificity was determined using primary (C1–C8) and secondary (C3–C6) alcohols (60 mM), diols and polyols (60 mM), or aldehydes and ketones (30 mM) under optimal enzymatic conditions determined.

Kinetic parameters of HbADH2 were determined using various substrates and coenzymes [NADP^+^ or NADPH]. For the oxidation reaction, 1‐propanol, 2‐propanol, ethanol, and 1‐butanol were used, while for the reduction reaction, the corresponding ketones and aldehydes, including acetone, propanal, acetaldehyde, and butyraldehyde, were used to determine the respective values of *K*_m_ and *k*_cat_. Unless specified, various substrates with concentrations from 0 to ≥ 10x apparent *K*_m_ were used to determine the corresponding activities. The concentration of the corresponding cofactor [NADP^+^ or NADPH] was kept constant and ≥10× apparent *K*_m_. Apparent values of *K*_m_ and *k*_cat_ were calculated using the nonlinear curve fittings of the Michaelis–Menten equation from GraphPad Prism (GraphPad Software) $V=\frac{V\max[S]}{Km+[S]}$.

### Sequence analysis

HbADH2 was purified as described above and only one band was visible on an SDS‐PAGE. The band was precisely cut out and preserved in Millipore water in an Eppendorf tube wrapped in parafilm. It was then sent out for peptide identification by mass spectrometry at the Alberta Proteomics and Mass Spectrometry Facility, University of Alberta (AB, Canada). A 3‐D structure of the HbADH2 monomer was modeled and superimposed with ADH from *T. maritima* (TM0920; PDB number: 1O2D) using the Swiss Model server^21^.

### Protein determination

The Bradford assay was used to determine protein concentration in solutions using a spectrophotometer^22^. After adding 200 µl of Bio‐Rad reagent into 800 μl of diluted protein solution, they were mixed and incubated at room temperature before taking the absorbance reading at 595 nm. A control assay was carried out by replacing the protein solution with 800 μl of deionized water. A calibration curve was made using known concentrations of the standard protein bovine serum albumin (BSA, albumin fraction V).

## AUTHOR CONTRIBUTIONS

**Ching Tse**: Conceptualization (supporting); data curation (lead); formal analysis (equal); writing—original draft (lead); writing—review and editing (supporting). **Kesen Ma**: Conceptualization (lead); formal analysis (equal); funding acquisition (lead); investigation (equal); methodology (lead); project administration (lead); resources (lead); supervision (lead); writing—original draft (supporting); writing—review and editing (lead).

## ETHICS STATEMENT

No animal or human research was involved in this study.

## CONFLICT OF INTERESTS

The authors declare no conflict of interests.

## Supporting information

Supporting information.

## Data Availability

The authors confirm that the data supporting the findings of this study are available within the article and its supporting information materials.
