# Supplementary material for: A novel alcohol dehydrogenase in the hyperthermophilic crenarchaeon Hyperthermus butylicus
Source: mLife. 2024 Jun 28;3(2):317–25. doi: 10.1002/mlf2.12126 (PMC11211662; doi:10.1002/mlf2.12126)
Supplement: Supplementary file 1 — Supporting information. [file MLF2-3-317-s001.docx]

**Supporting information Section**

**A novel alcohol dehydrogenase in the hyperthermophilic crenarchaeon *Hyperthermus butylicus***

**Ching Tse**^[[1]](#footnote-1)^ **and Kesen Ma**^1,*^

**Table S1**. Purification of the novel HbADH2 from *H. butylicus*

|  | Total protein  (mg) | Total activity (U) | Specific activity (U/mg) | Purification fold | Yield (%) |
| --- | --- | --- | --- | --- | --- |
| CFE | 30 | 41 | 1.367 | 1.0 | 100 |
| DEAE-Sepharose | 23 | 36 | 1.565 | 1.1 | 88 |
| HAP | 5.1 | 29 | 5.854 | 4.3 | 71 |
| phenyl-Sepharose | 2.6 | 21 | 8.236 | 6.0 | 52 |

**Table S2**. Mass spectrometry data with peptides from *H. butylicus*

| **Description** | **Score^a^** | **Coverage^b^** | **Proteins^c^** | **Unique Peptides^d^** | **Peptides^e^** |
| --- | --- | --- | --- | --- | --- |
| HBUT_RS04850 | 767.9 | 75.36 | 1 | 29 | 29 |
| HBUT_RS04595 | 69.51 | 39.21 | 1 | 13 | 13 |
| HBUT_RS08315 | 60.67 | 46.49 | 1 | 13 | 13 |
| HBUT_RS02620 | 16.88 | 16.55 | 1 | 6 | 6 |
| HBUT_RS02345 | 13.33 | 18.98 | 1 | 5 | 5 |

Note: a) Protein score is the sum of the ion scores of all peptides that were identified. b) Coverage is the percentage of the protein sequence covered by identified peptides. c) proteins represent the number of identified proteins in a protein group. d) unique peptides represent the number of peptide sequences that are unique to a protein group. e) peptides represent the total number of distinct peptide sequences identified in the protein group.

**Table S3.** Proteins with the highest similarities with the amino acid sequence of *H. butylicus* HbADH2

| **Description** | **E-value** | **% identification** | **Accession** |
| --- | --- | --- | --- |
| hypothetical protein of *Pyrodictium delaneyi* | 1e^-36^ | 33.82% | HIQ23459.1 |
| hypothetical protein of *Pyrodictium occultum* | 4e^-36^ | 33.52% | WP_058370604.1 |
| hypothetical protein of *Pyrodictium delaneyi* | 4e^-31^ | 30.46% | WP_088171899.1 |
| hypothetical protein of *Pyrodictium delaneyi* | 4e^-31^ | 30.06% | WP_055409879.1 |

Note: The proteins showed are with the highest percentage of identity with the amino acid sequence encoded by HBUT_RS04850 from the BLASTP program (protein to protein blast) using the database of all non-redundant GenBank CDS (nr) available from BLASTP 2.14.1+^1^.

**Figure S1.** Protein concentration dependency of *H. butylicus* HbADH2 activity. Enzyme assays were performed using 100 mM 4-(2-Hydroxyethyl)-1-piperazine propanesulfonic acid (EPPS) at pH 9.0 with 60 mM of 1-butanol and 0.4 mM of NADP^+^ at 80°C, the respective reaction was initiated with different amounts of purified HbADH2. Data were presented as mean ± standard deviation (n=2).

y = -0.007256x + 4.580
R² = 0.654

y = -0.02886x + 4.543
R² = 0.942

**Figure S2.** Thermostability of *H. butylicus* HbADH2. The residual activities (in the natural log of respective percentages of remaining activities) were plotted against the time of incubation at 85°C (filled circles) and 95°C (filled triangles). Data were presented as mean ± standard deviation (n=2). Inactivation rates at 85°C (filled circles) and 95°C (filled triangles) were calculated from the curve-fitting equations (B).

y = -0.05242x + 4.552
R² = 0.9945

**Figure S3.** Oxygen sensitivity of *H. butylicus* HbADH2. The residual activities (in the natural log of respective percentages of remaining activities) were plotted against time of exposure to air. Data were presented as mean ± standard derivation (n=2). Inactivation rate was calculated from the curve-fitting equation (B).

**Figure S4**. Comparison of amino acid sequences of HbADH2 encoded by HBUT_RS04850 and other characterized iron-containing ADHs. The sequences were aligned using Analysis Tool Web Services, Clustal Omega, from the EMBL-EBI^2^. HbADH, amino acid sequence encoded by HBUT_RS04850 from *H. butylicus* (WP_011822090.1); TnADH: ADH from *Thermotoga* *neapolitana* DSM 4359(ACM22756.1); ThADH: ADH from *Thermococcus hydrothermalis* (CAA74334.1); TzADH, ADH from *Thermococcus zilligii* AN1 (AAB63011.1); TlADH, ADH from *Thermococcus litoralis* (EHR78112.2); TpADH, ADH from *Thermococcus paralvinellae* ES-1 (ACK56133.1). “*”, amino acid residues that are identical in all sequences in the alignment; “:”, conserved substitutions; “.”, semi-conserved substitutions; “-”, no corresponding amino acid. Highlighted in grey, the conserved putative motif of cofactor binding sites; Highlighted in yellow, the conserved putative motif of iron binding sites. Red: small and hydrophobic amino acids; Blue: acidic amino acids; Magenta: basic amino acids excluding histidine; Green: amino acids with hydroxyl, sulfhydryl or amine group and glycine.

**References**

1. Altschul, S.F., Madden, T.L., Schäffer A.A., Zhang, J., Zhang, Z., Miller, W., et al. (1997). Gapped BLAST and PSI-BLAST: a new generation of protein database search programs, *Nucleic Acids Res.* *25*(17), 3389-3402.
2. Sievers, F., Wilm, A., Dineen, D., Gibson, T.J., Karplus, K., Li, W., et al (2011). **Fast, scalable generation of high-quality protein multiple sequence alignments using Clustal Omega, *Mol Syst Biol.* *7*(1), 539-544.**

1. Department of Biology, University of Waterloo, Waterloo, Ontario, Canada

   ^*^ **Correspondence**: Kesen Ma, kesen.ma@uwaterloo.ca [↑](#footnote-ref-1)
